# Comparison between Flow Cytometry and Traditional Culture Methods for Efficacy Assessment of Six Disinfectant Agents against Nosocomial Bacterial Species

**DOI:** 10.3389/fmicb.2017.00112

**Published:** 2017-02-03

**Authors:** Richard Massicotte, Akier A. Mafu, Darakhshan Ahmad, Francis Deshaies, Gilbert Pichette, Pierre Belhumeur

**Affiliations:** ^1^Centre Intégré de Santé et de Services Sociaux de LanaudièreQuébec, QC, Canada; ^2^Food Research and Development Centre, Agriculture and Agri-Food CanadaSt-Hyacinthe, QC, Canada; ^3^Hôpital du Sacré-Coeur de MontréalMontréal, QC, Canada; ^4^Department of Microbiology, Infectiology and Immunology, University of Montreal, Edouard-MontpetitMontreal, QC, Canada

**Keywords:** flow cytometry, disinfectant, viability status profiling, dead and live in between cell populations, nosocomial infections, healthcare facilities, minimum lethal concentrations

## Abstract

The present study was undertaken to compare the use of flow cytometry (FCM) and traditional culture methods for efficacy assessment of six disinfectants used in Quebec hospitals including: two quaternary ammonium-based, two activated hydrogen peroxide-based, one phenol-based, and one sodium hypochlorite-based. Four nosocomial bacterial species, *Escherichia coli, Staphylococcus aureus, Pseudomonas aeruginosa*, and Vancomycin-resistant *Enterococci faecalis*, were exposed to minimum lethal concentrations (MLCs) and sublethal concentrations (1/2 MLCs) of disinfectants under study. The results showed a strong correlation between the two techniques for the presence of dead and live cell populations, as well as, evidence of injured populations with the FCM. The only exception was observed with sodium hypochlorite at higher concentrations where fluorescence was diminished and underestimating dead cell population. The results also showed that FCM can replace traditional microbiological methods to study disinfectant efficacy on bacteria. Furthermore, FCM profiles for *E. coli* and *E. faecalis* cells exposed to sublethal concentrations exhibited distinct populations of injured cells, opening a new aspect for future research and investigation to elucidate the role of injured, cultural/noncuturable/resuscitable cell populations in infection control.

## Introduction

Surface disinfectants play a major role in controlling the environmental transmission and spread of nosocomial infections by contact with hitherto “noncritical” inanimate/environmental surfaces in the hospital and healthcare facilities (Mafu et al., 2015). In an effort to evaluate their comparative efficacies and specificities against different groups of pathogens, we conducted a culture-based study using *in vitro* germicidal exposure method and reported our findings recently (Deshaies et al., 2012). Using six disinfectants most commonly used in hospitals and healthcare facilities in Quebec at their minimum lethal concentrations (MLCs), defined as the lowest disinfectant concentration at which the bacterial cells were killed (Deshaies et al., 2012), we found in an *in vitro* germicidal assay that the choice of disinfectant agents and the hospital decontamination protocols can markedly affect the prevalence and environmental distribution of pathogens.

It was concluded that this could be better managed if a proper assessment of the risk associated with the use of disinfectants at off-recommended strengths conditions is considered to provide guidance toward and seeking satisfactory solutions (Deshaies et al., 2012). In such *in vitro* efficacy studies targeted to develop guidelines for on-site/*in vivo* practices, the action to be taken is by nature delayed to get the results by traditional/ classical culture based methods. There is a constant effort to develop and validate rapid methods to evaluate the efficacies of disinfectants. In this attempt, a rapid, yet reliable and robust technique could give an insight into one of the most challenging current issues in microbiological methods. Rapid DNA-based techniques do tell us the presence and identity of the microorganisms in the sample, but cannot give a good representation of the dead/live status of the cells, hindering their application as substitute to classical culture-based techniques. Flow cytometry (FCM) combined with viability staining using propidium iodine (PI) marker to assess the integrity of the cell membrane is a suitable technique for this kind of study (Davey and Kell, 1996; Fugère et al., 1996; Nebe-von-Caron et al., 1999; Wozniak-Kosek and Kawiak, 2005; Allegra et al., 2008; Cronin and Wilkinson, 2010; Hwang et al., 2012).

In current study, we compared the classical microbiological techniques (spot plating and staining and microscopy) with FCM for more in depth knowledge of the composition of the culture before and after exposure to the disinfectants, beside live (cfu) and dead, as well as injured/in-between subpopulations that may be culturable/resuscitable or non-cuturable/non-resuscitable (Holah et al., 1998; Tokashiki et al., 2007; Langsrud and Sundheim, 2008). FCM-based methods have advantage of short time indication of the microbial mortality level in the sample exposed to disinfectants, in contrast to classical culture-based method that take a minimum of 24 h. The cytometry in flow was already used successfully in different study using products disinfectants (Borazjani et al., 2000; Langsrud and Sundheim, 2008). However, there are very few studies which compare the use of the classic technique and the cytometry for the study of disinfectants as the one who was realized by Wozniak-Kosek and Kawiak (2005). The six disinfectants used in this study, chosen to represent different active pharmaceutical ingredients namely: quaternary ammoniums (QA)-, hydrogen peroxide-, phenol-, and chlorine-based agents. All disinfectants were tested based on manufacturers recommendations (lethal) and various off/-beyond recommended concentrations (s) (MLCs and sublethal). The present study is the first one which highlights a problem concerning the use of cytometry to study effect of the hypochlorite of sodium on bacterial colonies. Furthermore, we did not find any study showing that the use of minimal lethal concentration can produce subpopulations of wounded bacteria.

## Materials and methods

### Bacterial isolates, growth and sporulation media and culture conditions

Bacterial isolates used in this study were *Staphylococcus aureus* (ATCC no. 25923), *Enterococcus faecalis* (ATCC no. 19453), *Escherichia coli* (ATCC no. 25922) and *Pseudomonas aeruginosa* (ATCC no. 27853). The culture media and growth conditions were as described in NF EN 1040 and NF EN 14347 with modifications to adapt the technique to a microscale level as described previously (Deshaies et al., 2012).

Precultures were prepared from stock cultures stored at −80°C by streaking on appropriate media plates, then inoculating into liquid Tryptic Soy Broth (TSB) media and incubation aerobically at 37°C. The cultures were grown overnight (18–24 h) in liquid TSB media, harvested by centrifugation, washed three times, resuspended in Tryptone-buffer to an OD_620_ of ~0.1 (~5 × 10^8^ cell/mL) measured using a spectrophotometer and subsequently used for other bactericidal efficacy assays.

### Disinfectants tested

Six commonly used antimicrobial products of commercial grade from four different groups of active ingredients representing different classes of disinfectants (two quaternary ammonium (QA)-based, two hydrogen-peroxide-based, one phenol-based, one chlorine-based) sold in concentrated dosage forms, were obtained from local hospitals distributors. The different dilutions were made from the concentration recommended by the manufacturers. The MLCs against different bacterial species for each disinfectant product used in this study were determined in previous study as shown in Table 1 (Deshaies et al., 2012).

**Table 1:** **The minimum lethal concentrations (MLCs) against different bacterial species for disinfectant products used in this study**.

**Bacterial species**	**Disinfectant products**	**Manufacturer recommended concentrations (mg/L)**	**MLCs of the product (mg/L)**	**Disinfectant efficacy fold dilution required from manufacturer recommended concentrations to achieve MLCs**
*E. coli*	Phenol based	380:100	48:13	8x
	Activated hydrogen peroxide- based bactericide	3125	95	16x
	Activated hydrogen peroxide- based tuberculocide	5000	1563	32x
	Quat amm 4th	1184	19	64x
	Quat amm 3rd	360:360	6:6	64x
	Chlorine based	5250	82	64x
*E. faecalis*	Phenol based	380:100	48:13	8x
	Activated hydrogen peroxide- based bactericide	3125	98	32x
	Activated hydrogen peroxide- based tuberculocide	5000	78	64x
	Quat amm 4th	1184	19	64x
	Quat amm 3rd	360:360	6:6	64x
	Chlorine based	5250	164	32x
*P. aeruginosa*	Phenol based	380:100	48:13	8x
	Activated hydrogen peroxide- based bactericide	3125	98	32x
	Activated hydrogen peroxide- based tuberculocide	5000	78	64x
	Quat amm 4th	1184	9	128x
	Quat amm 3rd	360:360	3:3	128x
	Chlorine based	5250	82	64x
*S. aureus*	Phenol based	380:100	48:13	8x
	Activated hydrogen peroxide- based bactericide	3125	49	64x
	Activated hydrogen peroxide- based tuberculocide	5000	39	128x
	Quat amm 4th	1184	19	64x
	Quat amm 3rd	360:360	6:6	64x
	Chlorine solution	5250	82	64x

### Microbicidal efficacy assay conditions

Bactericidal efficacy test assays for disinfectants studied were performed at MLC/MLCs (presented in Table 1) concentrations, essentially following the protocols described in NF EN1040 (Borazjani et al., 2000; NF EN 1040 (T 72–152), 2005; Wozniak-Kosek and Kawiak, 2005; Langsrud and Sundheim, 2008) and NF EN 14347 (NF EN 14347, 2005) albeit adapted to a microscale level using a 96-well microtiter plate as described previously (Mafu et al., 2015). Each assay mix contained 20 μL of bacterial suspension, 20 μL of water and 160 μL of the disinfectant. After the predetermined contact time of 5 min at 20°C *(room temperature)*, 20 μL of the assay mix was transferred to the second row of wells of the plate containing 20 μL of water and 160 μL of an FCM compatible BD neutralizing buffer (cat no. 220518, BD Biosciences). After 5 min of neutralization, the treated bacterial suspensions were sampled and checked for viability either by the plate count method or FCM using fluorescent microscopy. Untreated controls were included for all assay setups. At the time the study was performed the assay media used conformed to Health Canada Standard: D-01.010R-70/General disinfectant claims/AOAC use Dilution.

### Viability state assay by classical plate count method (live/dead cell populations)

At the end of neutralization period, aliquots of 5 μL of the assay mix were spot plated onto Tryptic Soy Agar (TSA) plates and incubated for 24 h at 37°C under required conditions. For initial bacterial counts, serial dilutions of the initial bacterial suspension were plate on TSA and incubated under required conditions. Colonies forming units (cfu) were then recorded and the log reduction values were determined to evaluate the bactericidal efficacy under different assay setups. The assays were done in duplicate and repeated three times.

### Viability state profiling by flow cytometry (FCM) and fluorescent microscopy

To assess the viability status of bacterial cells in neutralized assay mix by FCM, aliquots of 20 μL were transferred into tubes containing 500 μL PBS. Thereafter, 10 μL of the dye reagent solution containing PI (cat no. 349483, BD Biosciences, City, State, and Country) was added to stain damaged cells. After at least 5 min of incubation with PI, the samples were analyzed quantitatively for dead, live and injured cell by FCM (FACSCalibur, BD Bioscience, City, State, and Country), and observed by fluorescence microscopy (Alsharif and Godfrey, 2002).

## Results

### Validation of viability profiling method by flow cytometry (FCM) compared to fluorescent microscopy and classical spot-plating using *E. coli* cells exposed to QA-based disinfectant

**Figure 1:**
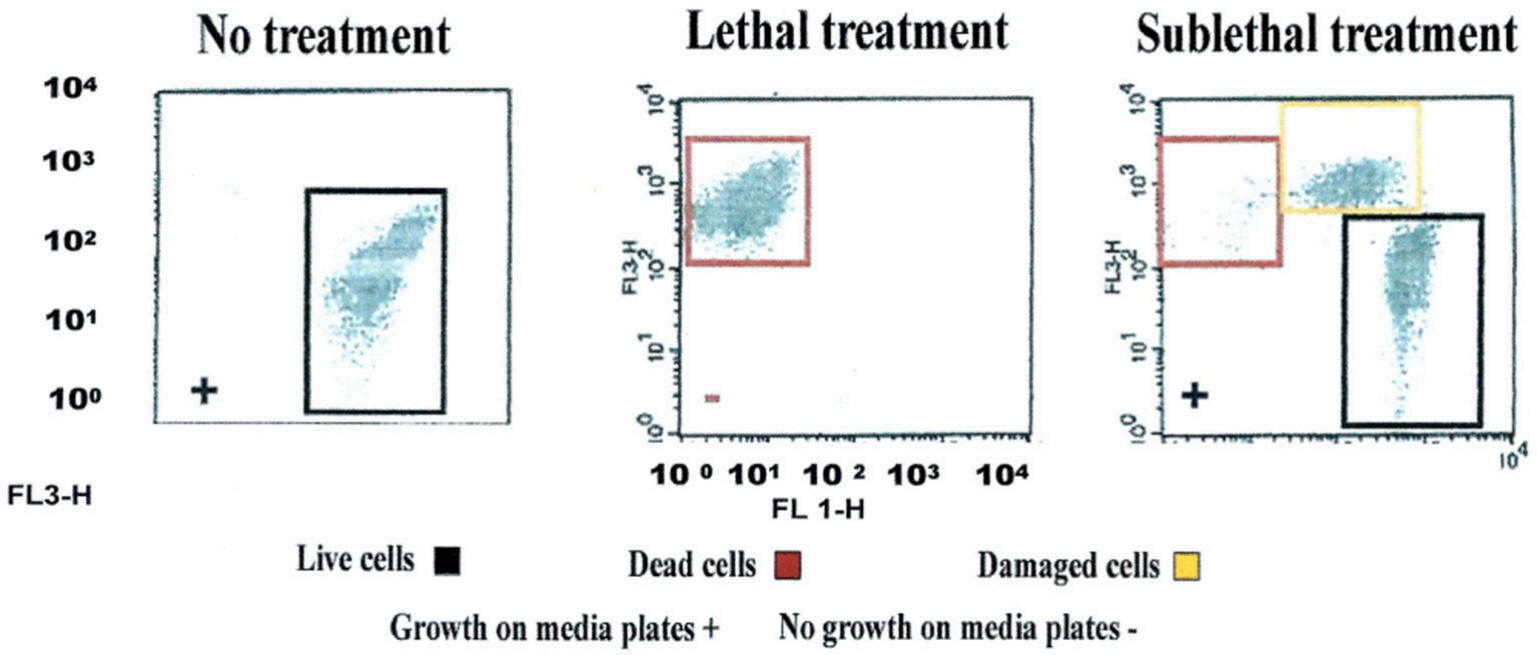
**Flow cytometric (FCM) analysis of viability profiles in population of ***E. coli*** cells exposed to a QA-based disinfectant (quaternary 4th generation) and stained with PI at minimum lethal and sublethal concentration (1/4 MLC)**. Light-scattering profile of the bacterial suspension: FS, forward scatter; SS, side scatter; Fluorescence signal FL1 (SS) and FL3 (FS). The bacterial populations were gated using FSC vs. SSC dot plot. Regions set around dead, live and injured/in-between populations were discriminated using FL1 vs. FL3 dot plot gated by scatter. (Growth of cells exposed to the disinfectant on media plates is embedded in the plots (as positive, +, negative, −).

**Figure 2:**
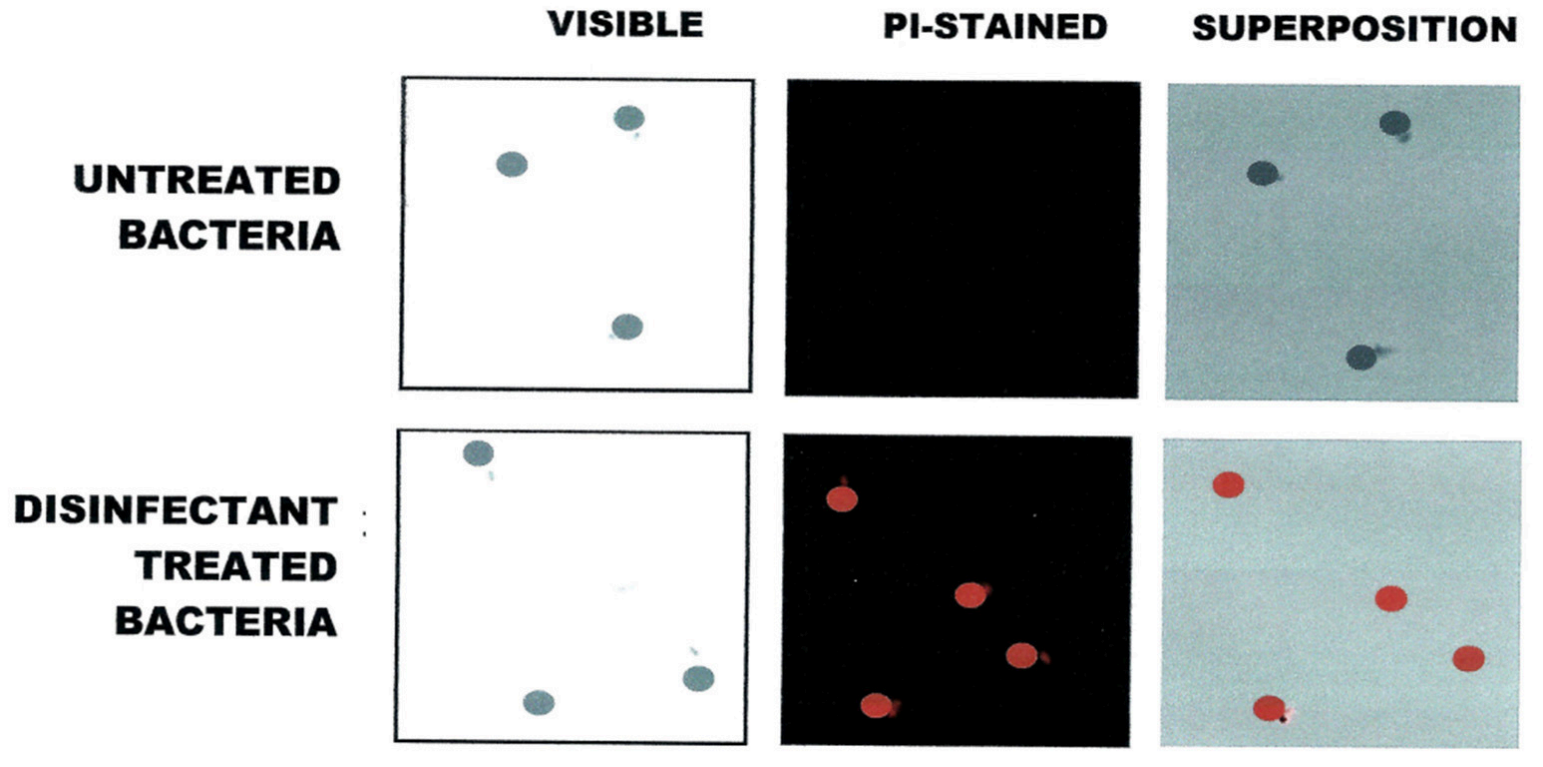
**Fluorescence Microscopic visualization of ***E. coli*** cells exposed to QA-based disinfectant and stained with PI (in red)**. Cells were treated with QUAT 4th generation at manufacturer recommended lethal concentration.

Validation of viability state profiling method used for the study was done using *E. coli* cells treated with a QA-based disinfectant (quaternary 4th generation) at minimum lethal and sublethal concentration (see Table 1) as determined by Deshaies et al. (2012). Viability state analysis by FCM (Figure 1) and fluorescent microscopy (Tx Red setting; Figure 2) were done on treated and untreated samples and the results were compared with those from the plate count (spot-plating on agar media). Gating and analysis strategy for front scatter counts (FSC) vs. side scatter counts (SSC) dot plots, presented in Figure 1, shows that all cells in the sample treated with lethal concentration of the disinfectant contained only one population of cells stained with PI (dead cells) mapping on the left upper quadrant region (Red zone representing dead cell area). Cells in the untreated sample contained only one population of unstained cells (live cells, control) mapping distinctly on the right lower quadrant region (Black zone representing the live cell area). Interestingly, FCM profile from the sample treated with MLCs of disinfectant showed a 1/3 distinct populations of cells located between live and dead cells region (Orange zone on Figure 1). Fluorescent microscopy of the two types of samples, treated at manufacturer's recommended (lethal) concentration, presented in Figure 2, corroborated with the results from FCM for dead and live cells. Similarly, samples from untreated control (live control) and sublethal treatment showed good growth on agar media whereas no growth was seen with samples from lethal treatment (dead control), validating the results from the FCM (Figure 1).

### Validation of viability profiling method by flow cytometry (FCM) compared to classical spot-plating using *E. coli, P. aeruginosa, E. faecal*, and *S. aureus* cells exposed to various disinfectants

**Figure 3:**
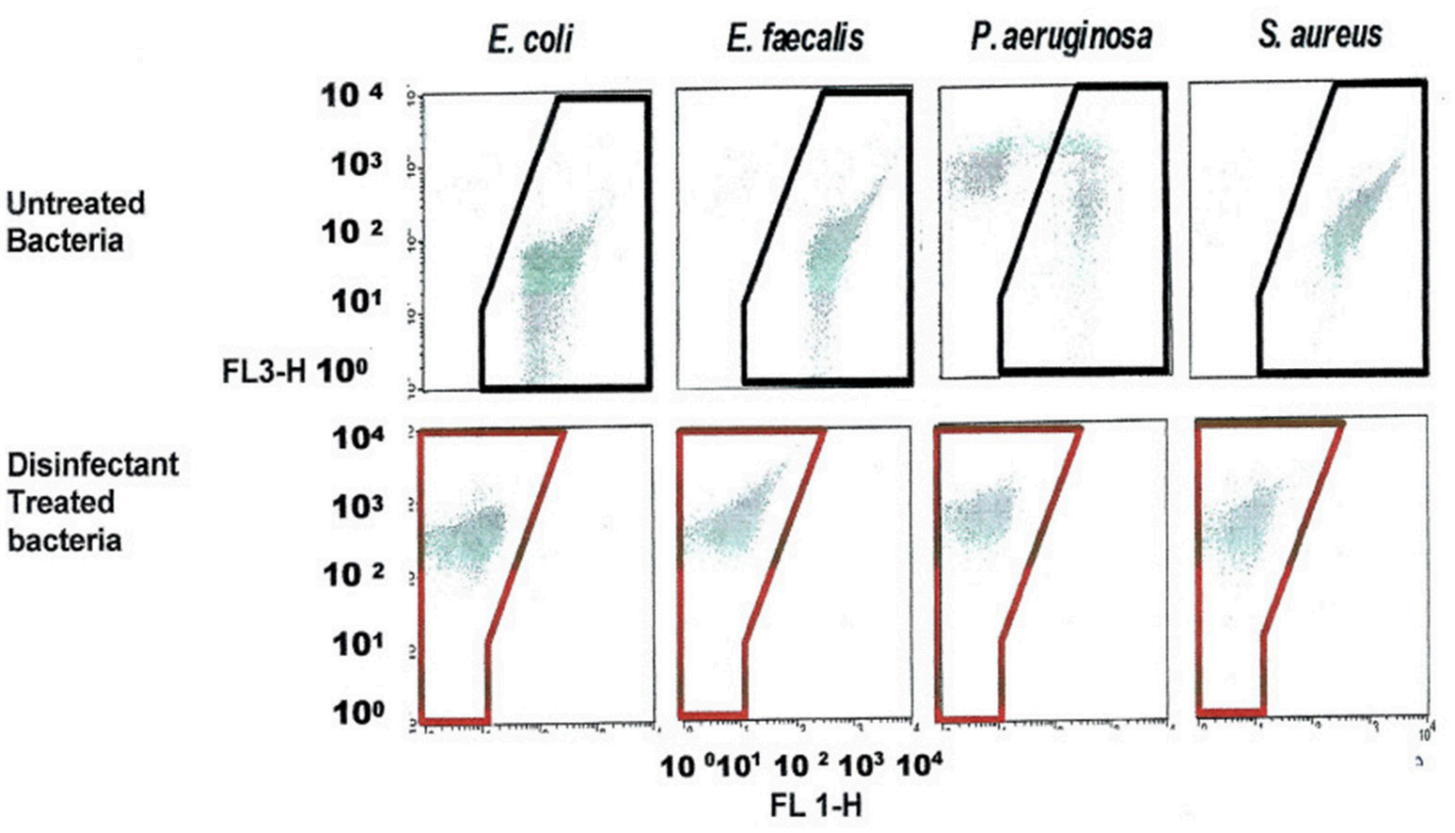
**FCM dot plots with Gating zones for bacterial populations and analysis strategy used to evaluate the viability (dead in red zone, live in a black zone) of treated and untreated bacterial cell with a QA-based disinfectant (quaternary 4th generation) stained with PI**. The bacterial populations were gated using FSC vs. SSC dot plots. Dead, live and injured/in-between populations were discriminated using FL1 vs. FL3.

FCM scatter plot obtained for cells treated at manufacturer recommended concentrations (MRCs) for QA 4th generation, and untreated cells, on four bacterial strains, including *E. coli*, and the gating/zoning and analysis strategy used to designate dead cell control (red zone) and live cell control (black zone) cell areas are presented in Figure 3. In order to define these areas, a population of dead *E. coli* cells treated with a QA-based disinfectant and stained with PI was adjusted to fit between 10^2^ and 10^3^ in the FL3-axis, and between 10^0^ and 10^1^ in the FL1-axis. All other samples (live *E. coli* as well as any other bacteria, dead or alive) were analyzed without any further adjustment. A “universal” gating zone for living cells as well as dead cells was then defined (see Figure 3). To be considered alive, >1% of the total number of cells must be present in the live gating. This cut-off value prevents background signal to generate false positive results.

**Figure 4:**
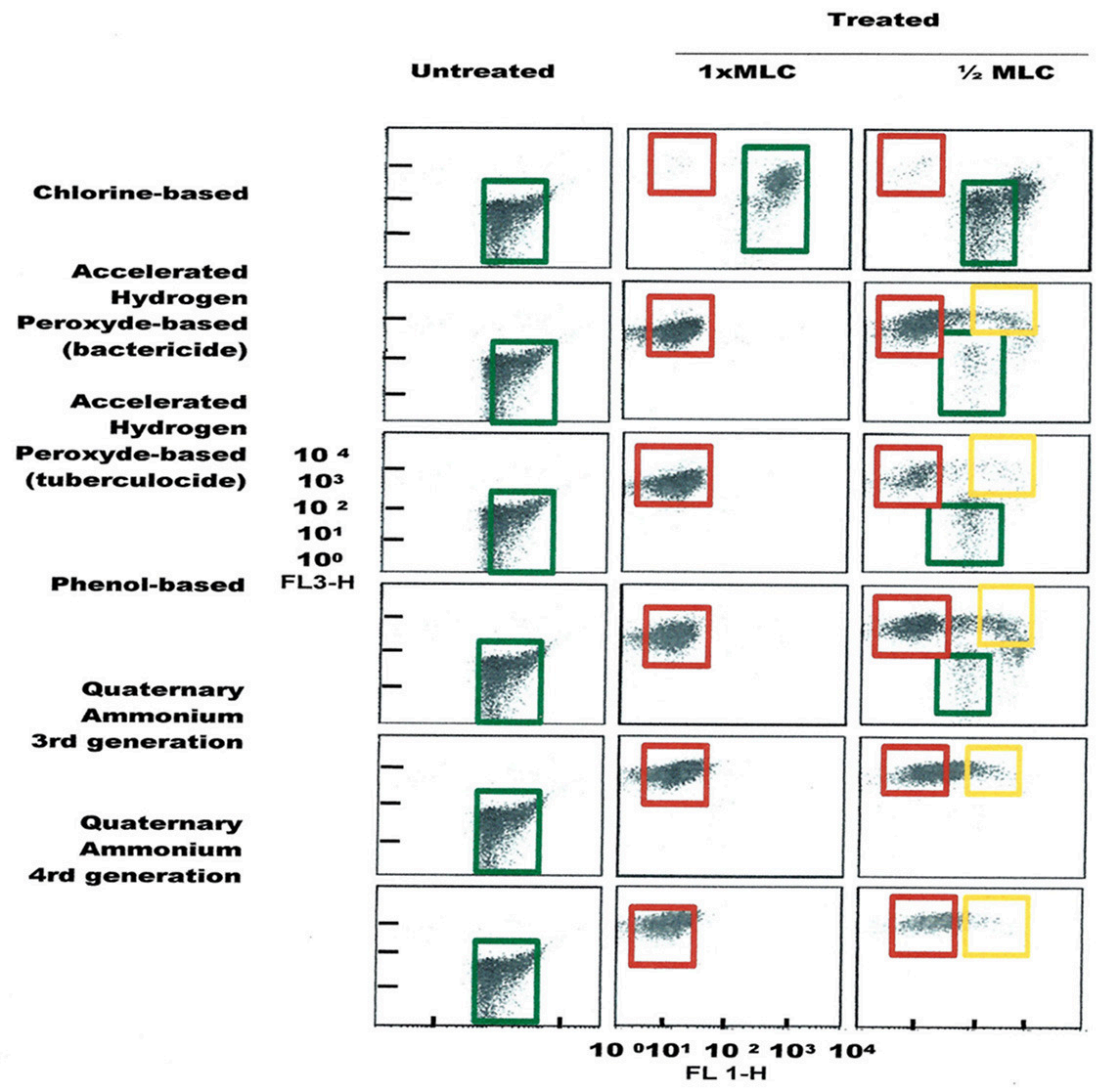
**FCM dot plots for ***E. coli*** cells exposed to different types of disinfectants at minimum lethal and sublethal concentrations**. Quadrant zones: Green, live cells; Red, dead cells; Orange, damaged/injured cells.

**Figure 5:**
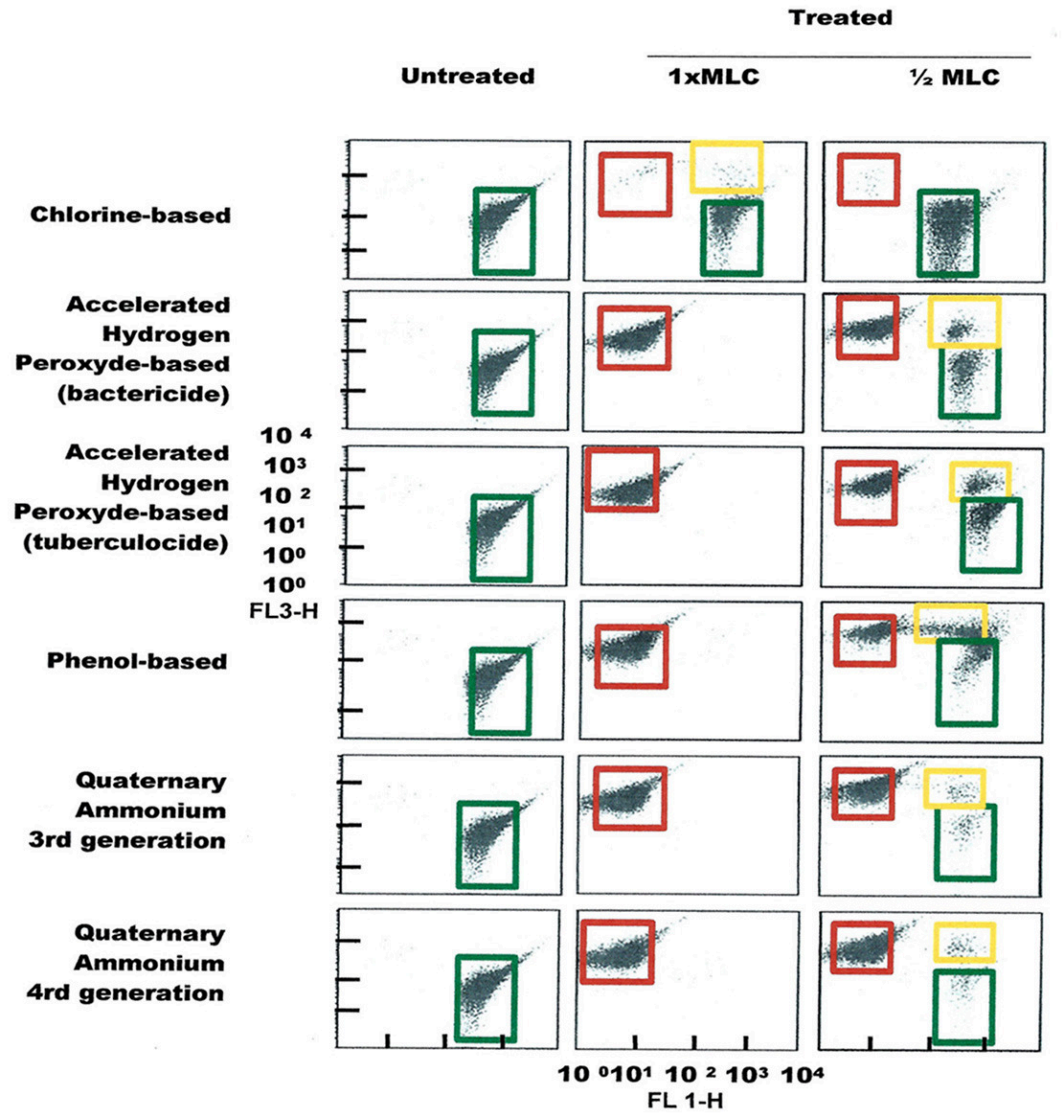
**FCM dot plots for ***E. faecalis*** cells exposed to different types of disinfectants at minimum lethal and sublethal concentrations**. Quadrant zones: Green, live cells; Red, dead cells; Orange, damaged/injured cells.

**Figure 6:**
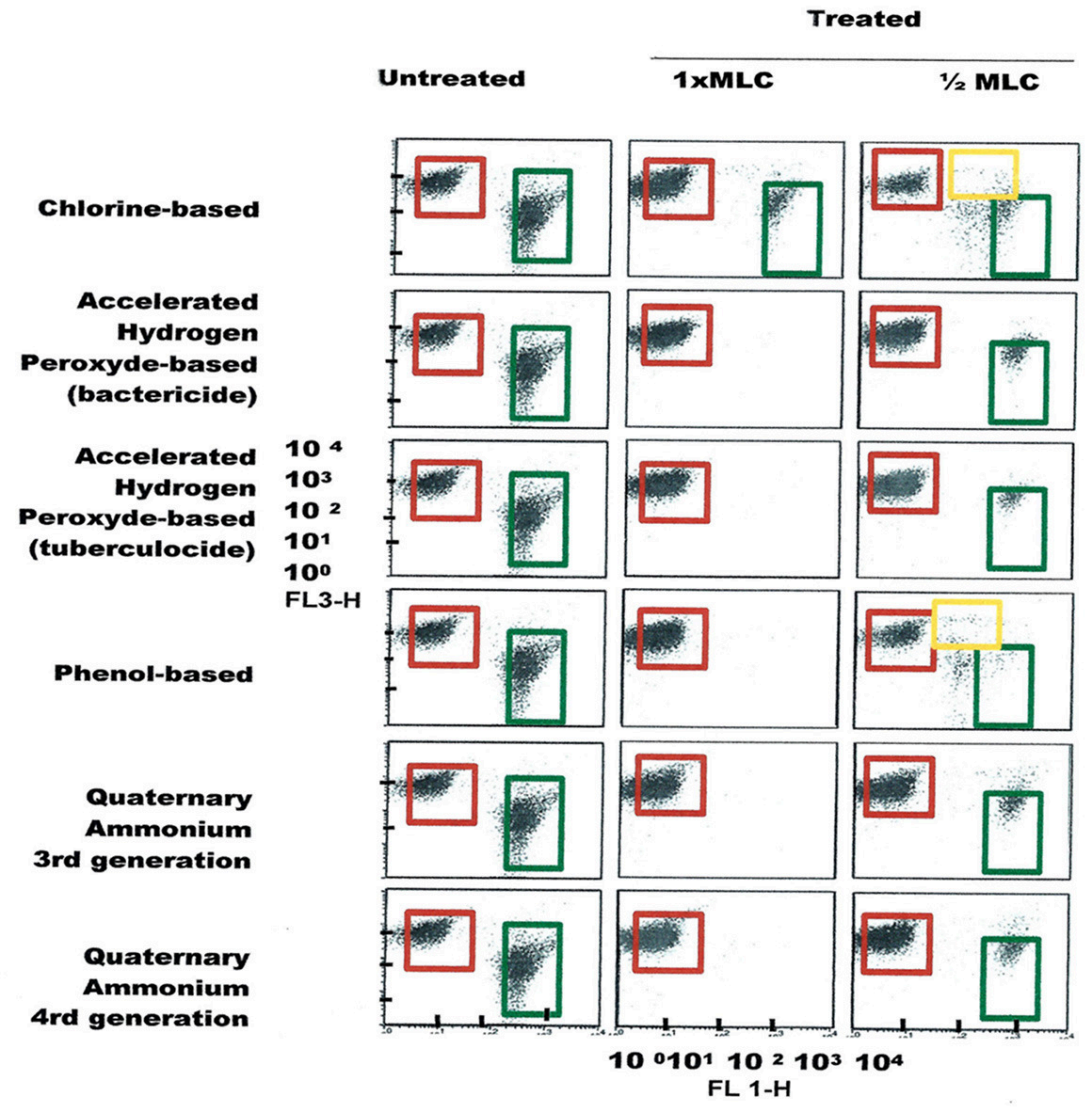
**FCM dot plots for ***P. aeruginosa*** cells exposed to different types of disinfectants at minimum lethal and sublethal concentrations**. Quadrant zones: Green, live cells; Red, dead cells; Orange, damaged/injured cells.

**Figure 7:**
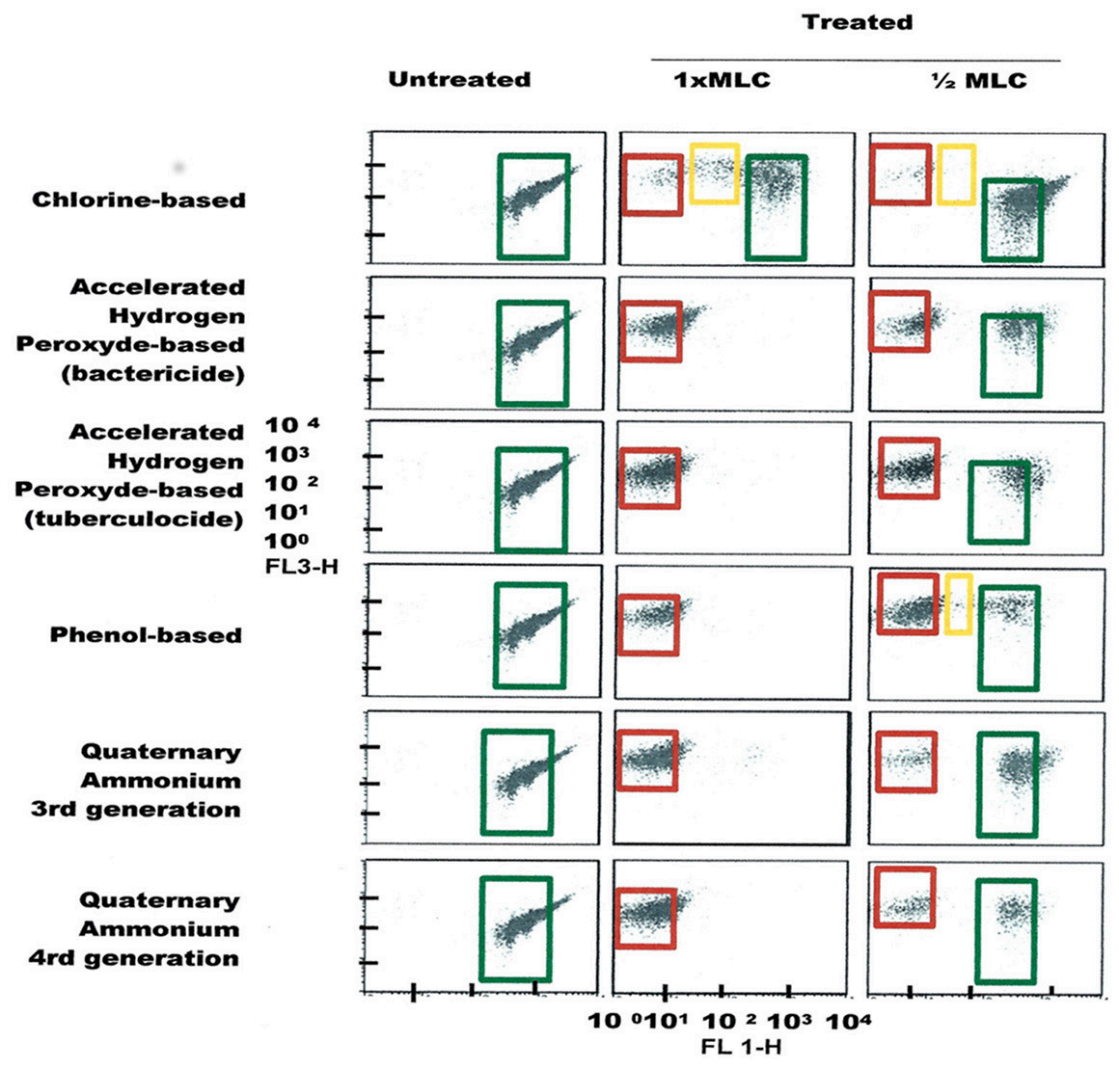
**FCM dot plots for ***S. aureus*** cells exposed to different types of disinfectants at minimum lethal and sublethal concentrations**. Quadrant zones: Green, live cells; Red, dead cells; Orange, damaged/injured cells.

FCM profiles obtained for the four test strains exposed to lethal and MLCs and 1/2MLCs of four different groups of disinfectants (chlorine-, hydrogen peroxide-, phenol- and QA-based), are presented in Figures 4–7. Besides the dead and live cell populations, a distinct zone of damaged/injured cells (orange zone) was observed for cells exposed to MLCs of all four disinfectants with all four strains tested. It should be noted that a significant part of untreated *P. aeruginosa* cell culture were dead before treatment.

Results obtained from FCM profiling were compared to culture-based classical methods, as summarized in Tables 2–5. The statistical analysis is presented in Table 6.

**Table 2:** **Comparison of viability status profiling results from FCM with classical culture-based technique (Micro) for ***E. coli*** treated with various disinfectants**.

**Bacteria**	**Product**	**No. trials**	Dilution^*^	**FCM**	**Micro**	**Positive tests (+) FCM**	**Positive test (+) MICRO**
***E. coli***	Sodium Hypochloride	1	1/64	+	−	7/7	5/7
		2	1/64	+	−		
		3	1/128	+	+		
		4	1/128	+	+		
		5	1/256	+	+		
		6	1/256	+	+		
		Ctrl	−	+	+		
	Accelerated hydrogen Peroxide based (bactericide)	1	1/16	−	−	4/7	4/7
		2	1/16	−	−		
		3	1/16	−	−		
		4	1/32	+	+		
		5	1/32	+	+		
		6	1/32	+	+		
		Ctrl	−	+	+		
	Accelerated hydrogen Peroxide based (tuberculocide)	1	1/32	−	−	4/7	4/7
		2	1/32	−	−		
		3	1/32	−	−		
		4	1/64	+	+		
		5	1/64	+	+		
		6	1/64	+	+		
		Ctrl	−	+	+		
	Phenol based	1	1/8	−	−	4/7	4/7
		2	1/8	−	−		
		3	1/8	−	−		
		4	1/16	+	+		
		5	1/16	+	+		
		6	1/16	+	+		
		Ctrl	−	+	+		
	Quaternary ammonium 3rd generation	1	1/32	−	−	4/8	4/8
		2	1/64	−	−		
		3	1/64	−	−		
		4	1/64	−	−		
		5	1/128	+	+		
		6	1/128	+	+		
		7	1/128	+	+		
		Ctrl	−	+	+		
	Quaternary ammonium 4th generation	1	1/32	−	−	4/8	4/8
		2	1/64	−	−		
		3	1/64	−	−		
		4	1/64	−	−		
		5	1/128	+	+		
		6	1/128	+	+		
		7	1/128	+	+		
		Ctrl	−	+	+		

*+, Live cells; −, Dead cells; Ctrl, Without disinfectant*.

* *Dilution performed further to manufacturer recommended working dilution*.

**Table 3:** **Comparison of viability status profiling results from FCM with classical culture-based technique (Micro) for ***E. faecalis*** treated with various disinfectants**.

**Bacteria**	**Product**	**No. trials**	Dilution^*^	**FCM**	**Micro**	**Positive tests (+) FCM**	**Positive test (+) MICRO**
*E. faecalis*	Sodium Hypochloride	1	1/16	+	−	13/13	7/13
		2	1/16	+	−		
		3	1/16	+	−		
		4	1/32	+	−		
		5	1/32	+	−		
		6	1/32	+	+		
		7	1/64	+	+		
		8	1/64	+	−		
		9	1/64	+	+		
		10	1/128	+	+		
		11	1/128	+	+		
		12	1/128	+	+		
		Ctrl	−	+	+		
	Accelerated hydrogen Peroxide based (bactericide)	1	1/32	−	−	4/7	4/7
		2	1/32	−	−		
		3	1/32	−	−		
		4	1/64	+	+		
		5	1/64	+	+		
		6	1/64	+	+		
		Ctrl	−	+	+		
	Accelerated hydrogen Peroxide based (tuberculocide)	1	1/64	−	−	4/7	4/7
		2	1/64	−	−		
		3	1/64	−	−		
		4	1/128	+	+		
		5	1/128	+	+		
		6	1/128	+	+		
		Ctrl	−	+	+		
	Phenol based	1	1/8	−	−	4/7	4/7
		2	1/8	−	−		
		3	1/8	−	−		
		4	1/16	+	+		
		5	1/16	+	+		
		6	1/16	+	+		
		Ctrl	−	+	+		
	Quaternary ammonium 3rd generation	1	1/64	+	+	8/10	8/10
		2	1/64	−	−		
		3	1/64	−	−		
		4	1/128	+	+		
		5	1/128	+	+		
		6	1/128	+	+		
		7	1/256	+	+		
		8	1/256	+	+		
		9	1/256	+	+		
		Ctrl	−	+	+		
	Quaternary ammonium 4th generation	1	1/64	+	+	8/10	8/10
		2	1/64	−	−		
		3	1/64	−	−		
		4	1/128	+	+		
		5	1/128	+	+		
		6	1/128	+	+		
		7	1/256	+	+		
		8	1/256	+	+		
		9	1/256	+	+		
		Ctrl	−	+	+		

*+, Live cells; −, Dead cells; Ctrl, Without disinfectant*.

* *Dilution performed further to manufacturer recommended working dilution*.

**Table 4:** **Comparison of viability status profiling results from FCM with classical culture-based technique (Micro) for ***P. aeroginosa*** treated with various disinfectants**.

**Bacteria**	**Product**	**No. trials**	Dilution^*^	**FCM**	**Micro**	**Positive tests (+) FCM**	**Positive test (+) MICRO**
*P. aeroginosa*	Sodium Hypochloride	1	1/64	+	−	9/9	4/9
		2	1/64	+	−		
		3	1/64	+	−		
		4	1/128	+	−		
		5	1/128	+	+		
		6	1/128	+	−		
		7	1/256	+	+		
		8	1/512	+	+		
		Ctrl	−	+	+		
	Accelerated hydrogen Peroxyde based (bactericide)	1	1/32	−	−	5/7	5/7
		2	1/32	+	+		
		3	1/32	−	−		
		4	1/64	+	+		
		5	1/64	+	+		
		6	1/64	+	+		
		Ctrl	−	+	+		
	Accelerated hydrogen Peroxyde based (tuberculocide)	1	1/64	−	−	5/7	5/7
		2	1/64	+	+		
		3	1/64	−	−		
		4	1/128	+	+		
		5	1/128	+	+		
		6	1/128	+	+		
		Ctrl	−	+	+		
	Phenol based	1	1/2	−	−	5/7	5/7
		2	1/2	−	−		
		3	1/4	+	+		
		4	1/2	+	+		
		5	1/4	+	+		
		6	1/4	+	+		
		Ctrl	−	+	+		
	Quaternary ammonium 3rd generation	1	1/128	−	−	6/10	7/10
		2	1/128	−	−		
		3	1/128	+	+		
		4	1/256	+	+		
		5	1/256	+	+		
		6	1/256	−	−		
		7	1/512	+	+		
		8	1/512	+	+		
		9	1/512	−	+		
		Ctrl	−	+	+		
	Quaternary ammonium 4th generation	1	1/128	−	−	6/10	7/10
		2	1/128	−	−		
		3	1/128	−	−		
		4	1/256	+	+		
		5	1/256	+	+		
		6	1/256	−	+		
		7	1/512	+	+		
		8	1/512	+	+		
		9	1/512	+	+		
		Ctrl	−	+	+		

*+, Live cells; −, Dead cells; Ctrl, Without disinfectant*.

* *Dilution performed further to manufacturer recommended working dilution*.

**Table 5:** **Comparison of viability status profiling results from FCM with classical culture-based technique (Micro) for ***S. aureus*** treated with various disinfectants**.

**Bacteria**	**Product**	**No. trials**	Dilution^*^	**FCM**	**Micro**	**Positive tests (+) FCM**	**Positive test (+) MICRO**
*S. aureus*	Sodium Hypochloride	1	1/32	+	−	9/9	5/9
		2	1/64	+	−		
		3	1/64	+	−		
		4	1/64	+	−		
		5	1/128	+	+		
		6	1/128	+	+		
		7	1/128	+	+		
		8	1/256	+	+		
		Ctrl	−	+	+		
	Accelerated hydrogen Peroxyde based (bactericide)	1	1/64	−	−	4/7	4/7
		2	1/64	−	−		
		3	1/128	+	+		
		4	1/128	−	−		
		5	1/128	+	+		
		6	1/256	+	+		
		Ctrl	−	+	+		
	Accelerated hydrogen Peroxyde based (tuberculocide)	1	1/128	−	−	4/7	4/7
		2	1/128	−	−		
		3	1/128	−	−		
		4	1/256	+	+		
		5	1/256	+	+		
		6	1/256	+	+		
		Ctrl	−	+	+		
	Phenol based	1	1/8	−	−	5/7	5/7
		2	1/8	−	−		
		3	1/16	+	+		
		4	1/16	+	+		
		5	1/32	+	+		
		6	1/32	+	+		
		Ctrl	−	+	+		
	Quaternary ammonium 3rd generation	1	1/64	−	−	5/7	5/7
		2	1/64	−	−		
		3	1/64	+	+		
		4	1/128	+	+		
		5	1/128	+	+		
		6	1/128	+	+		
		Ctrl	−	+	+		
	Quaternary ammonium 4th generation	1	1/64	−	−	8/10	8/10
		2	1/64	−	−		
		3	1/64	+	+		
		4	1/128	+	+		
		5	1/128	+	+		
		6	1/128	+	+		
		7	1/256	+	+		
		8	1/256	+	+		
		9	1/256	+	+		
		Ctrl	−	+	+		

*+, Live cells; −, Dead cells; Ctrl, Without disinfectant*.

* *Dilution performed further to manufacturer recommended working dilution*.

**Table 6:** **Concordance and Chi-square analysis of results obtained from FCM and classical culture-based techniques according to each disinfectant**.

**Product**	**Bacteria**	**Positive tests FCM**	**Positive test MICRO**	**Concordance %**	**Chi-square test analysis**
Sodium Hypochloride	*E. coli*	10	8	55.3	Nonequivalent method
	*E. faecalis*	13	7		>95 probability
	*P. aeroginosa*	9	4		
	*S. aureus*	9	5		
Accelerated hydrogen Peroxyde based (bactericide)	*E. coli*	4	4	100	Equivalent method
	*E. faecalis*	4	4		>95 probability
	*P. aeroginosa*	5	5		
	*S. aureus*	4	4		
Accelerated hydrogen Peroxyde based (tuberculocide)	*E. coli*	4	4	100	Equivalent method
	*E. faecalis*	4	4		>95 probability
	*P. aeroginosa*	5	5		
	*S. aureus*	4	4		
Phenol based	*E. coli*	4	4	100	Equivalent method
	*E. faecalis*	4	4		>95 probability
	*P. aeroginosa*	5	5		
	*S. aureus*	5	5		
Quaternary ammonium 3rd generation	*E. coli*	4	4	97.1	Equivalent method
	*E. faecalis*	8	8		>95 probability
	*P. aeroginosa*	6	7		
	*S. aureus*	5	5		
Quaternary ammonium 4th generation	*E. coli*	4	4		Equivalent method
	*E. faecalis*	8	8	97.4	>95 probability
	*P. aeroginosa*	6	7		
	*S. aureus*	8	8		

## Discussion

The results obtained from this study clearly demonstrate that the FCM-based rapid analysis of profiling for viability status of bacterial cells in cultures exposed to disinfectants well corroborated, with those obtained from traditional culture methods. One of the advantages of the cytometer technique is the quick response (few min) compare to the standard (24 h). It also provides a quick process to evaluate the effect of disinfectant on a new bacteria nosocomial. This technique detects cells, regardless of their ability to survive or not, and provides an idea of the present bacterial load and those dead or under sublethal conditions. The other advantage in comparison with the traditional culture methods and PCR technique is the possibility to observe the sub-population bacteria injured and study the presence of Viable Bacteria Non-Culturable (Oliver-James, 2009).

The comparison on a purely economic basis between the classic technique and the cytometry in flow for the evaluation of a disinfectant clearly works in favor of the classic technique. However, use of cytometry becomes advantageous when there is an emergency situation and when there is a risk of distribution in the case of a new type of infections. We have to know quickly the type of disinfectant which would be adequate. For example, the spores of Clostridium difficile are resistant in most of disinfectants products. To act quickly and effectively, we can present more efficient on socioeconomic plan (saving time and/or reducing number of death). During the Clostridium difficile epidemic in Quebec in 2003 (incidence rate of 13.1/1000 admissions), it would have been important at the time to have a good technique of use with good disinfectants in order to Reduce the incidence of this infection rapidly^1^. This was not the case. In 2003, a single case of a symptomatic person having contracted *Clostridium difficile* in the hospital environment could spend 12,000$ Canadian dollars more in hospitalization fees^1^.

In comparison with fluorescence microscopy this approach is very advantageous because it requires only one adjustment device.

Thereafter, whatever the condition or type of bacteria coupled with an automated system (let's spend hundreds of tubes without being present), the method would be more effective.

These results obtained are in agreement with earlier work on bacterial populations exposed to disinfectants (Wozniak-Kosek and Kawiak, 2005). The only exception was sodium hypochlorite where all four bacterial populations exposures at concentrations higher than the MLCs (e.g., 1/16 dilution) did not show correlation between the standard microbiological method and FCM. This was due, mainly, to chemical interaction/interference of hypochlorite ions and cell components. Phe et al. (2007) observed similar fluorescence deterioration of *E. coli* cells exposed to sodium hypochlorite (bleach) at a concentration >3 μmol/L of Cl_2_. In fact, such alteration/ deterioration of fluorescence may lead to false interpretations as damaged cells that would normally be permeable to the fluorescent dye, PI would be impermeable due to inhibition by the bleach. The authors explained the absence of fluorescence as damage caused by chlorine reacting with nucleic acids, both *in vitro* and *in vivo*, resulting in a reduced fluorescence of the complex (nucleic acid + fluorochromes) stained with SYBR-II gold PI. More precisely, reaction of cells with HOCl ions does manifest such alteration in fluorescence (Venkobachar et al., 1977; Hawkins and Davies, 2002; Phe et al., 2005) as HOCl ions damage DNA and RNA as well as polynucleotides to form chloramines (Hawkins and Davies, 2002). Hypothetically, in addition to this reaction between nucleic acid and fluorochromes, there is a possibly an oxidative effect of chlorine on the PI, and this remains to be studied. It is also possible that the absence of fluorescence in PI is the result of combined effect of the two interactions: bleaching actions and formation of a nucleic acid-fluorochromes complex.

It was also observed that on the FCM plots the zone of damaged cell population lies between the dead and living cell zones, mainly with sublethal concentrations (½ MLCs). The presence of sublethal populations is variable for bacteria species and disinfectants. For instance, ammonium generally generates few of these populations compared to the other disinfectants while phenol generates this type of bacteria population all the time. With regards to the type of bacteria, *E. faecalis* generated sublethal population for all disinfectants except sodium hypochlorite. This result suggests the increased persistence of *E. faecalis* in sublethal state and involvement of nosocomial infections where the used disinfectant concentration is not the right one. It would be interesting to know if the cells in this zone of damaged cells resuscitated and grow under favorable conditions in some environmental conditions or at least retain their living properties and remain non-culturable. Hypothetically, the presence of such populations on a surface could be the basis of maintenance of a certain environmental biocharge of bacteria with potential to spread of nosocomial infections or food poisoning. Studies in this direction should be undertaken.

## Conclusion

FCM method of analysing viability profile of bacterial cultures, exposed to a variety of disinfectants corroborates, significantly, with classical culture-based methods. On the other hand, use of sodium hypochlorite with the cytometry requires caution in the interpretation of the results since based on the concentration used, under-estimates the actual number of dead cell populations. Furthermore, FCM provides an interesting insight into damaged bacterial cell populations, especially, when low concentrations of disinfectant products are present. FACS method could become an effective tool to investigate and determine the viability status of bacterial cells isolated from surfaces when they are not treated with sodium hypochlorite. It could also be used to characterize new disinfectants that do not release a significant amount of HOCl ions.

## Author contributions

Conceived, designed the experiments and generated and analyzed the data: RM. As correspondence author revised and contributed to the conception of the work and analyzed the data: AM. Conceived, designed the experiments and revised the paper: DA. Performed the experiments: FD. Conceived and designed the experiments and revised the paper: GP. Conceived and designed the experiments and supervised all the study: PB.

### Conflict of interest statement

The authors declare that the research was conducted in the absence of any commercial or financial relationships that could be construed as a potential conflict of interest.

## Footnotes

^1^Committee on the hospital-borne infections of Quebec. Notices scientist Prevention measures and control of the infections in the urgency Management of the biological risks and the health in the work in September, 2013, 113p.
